# Occurrence of BMAA Isomers in Bloom-Impacted Lakes and Reservoirs of Brazil, Canada, France, Mexico, and the United Kingdom

**DOI:** 10.3390/toxins14040251

**Published:** 2022-03-31

**Authors:** Safa Abbes, Sung Vo Duy, Gabriel Munoz, Quoc Tuc Dinh, Dana F. Simon, Barry Husk, Helen M. Baulch, Brigitte Vinçon-Leite, Nathalie Fortin, Charles W. Greer, Megan L. Larsen, Jason J. Venkiteswaran, Felipe Fernando Martínez Jerónimo, Alessandra Giani, Chris D. Lowe, Nicolas Tromas, Sébastien Sauvé

**Affiliations:** 1 Department of Chemistry, Université de Montréal, Montréal, QC H2V 0B3, Canada; safa.abbes@umontreal.ca (S.A.); sung.vo.duy@umontreal.ca (S.V.D.); gabriel.munoz@umontreal.ca (G.M.); quoc.tuc.dinh@umontreal.ca (Q.T.D.); df.simon@umontreal.ca (D.F.S.); 2 BlueLeaf Inc., Drummondville, QC J2B 5E9, Canada; bhusk@blue-leaf.ca; 3 Global Institute for Water Security, University of Saskatchewan, Saskatoon, SK S7N 3H5, Canada; helen.baulch@usask.ca; 4 LEESU, École des Ponts, Université Paris Est Créteil, 94000 Créteil, France; b.vincon-leite@enpc.fr; 5 National Research Council Canada, Energy, Mining, and Environment, Montréal, QC H4P 2R2, Canada; nathalie.fortin@cnrc-nrc.gc.ca (N.F.); charles.greer@cnrc-nrc.gc.ca (C.W.G.); 6 Faculty of Science, Wilfrid Laurier University, Waterloo, ON N2L 3C5, Canada; meg.larsen87@gmail.com (M.L.L.); jvenkiteswaran@wlu.ca (J.J.V.); 7 Instituto Politécnico Nacional, Mexico City 11340, Mexico; fjeroni@ipn.mx; 8 Department of Botany, Universidade Federal de Minas Gerais, Belo Horizonte 31270-901, MG, Brazil; agiani@ufmg.br; 9 Centre for Ecology and Conservation, University of Exeter, Exeter TR10 9FE, UK; c.lowe@exeter.ac.uk; 10 Department of Biological Sciences, Université de Montréal, Montréal, QC H2V 0B3, Canada; tromas.nicolas@gmail.com

**Keywords:** lake water, β-N-methyl-amino-l-alanine (BMAA), 2,4-diaminobutyric acid (DAB), N-(2-aminoethyl) glycine (AEG), trichloroacetic acid (TCA), temporal trends

## Abstract

The neurotoxic alkaloid β-N-methyl-amino-l-alanine (BMAA) and related isomers, including N-(2-aminoethyl glycine) (AEG), β-amino-N-methyl alanine (BAMA), and 2,4-diaminobutyric acid (DAB), have been reported previously in cyanobacterial samples. However, there are conflicting reports regarding their occurrence in surface waters. In this study, we evaluated the impact of amending lake water samples with trichloroacetic acid (0.1 M TCA) on the detection of BMAA isomers, compared with pre-existing protocols. A sensitive instrumental method was enlisted for the survey, with limits of detection in the range of 5–10 ng L^−1^. Higher detection rates and significantly greater levels (paired Wilcoxon’s signed-rank tests, *p* < 0.001) of BMAA isomers were observed in TCA-amended samples (method B) compared to samples without TCA (method A). The overall range of B/A ratios was 0.67–8.25 for AEG (up to +725%) and 0.69–15.5 for DAB (up to +1450%), with absolute concentration increases in TCA-amended samples of up to +15,000 ng L^−1^ for AEG and +650 ng L^−1^ for DAB. We also documented the trends in the occurrence of BMAA isomers for a large breadth of field-collected lakes from Brazil, Canada, France, Mexico, and the United Kingdom. Data gathered during this overarching campaign (overall, *n* = 390 within 45 lake sampling sites) indicated frequent detections of AEG and DAB isomers, with detection rates of 30% and 43% and maximum levels of 19,000 ng L^−1^ and 1100 ng L^−1^, respectively. In contrast, BAMA was found in less than 8% of the water samples, and BMAA was not found in any sample. These results support the analyses of free-living cyanobacteria, wherein BMAA was often reported at concentrations of 2–4 orders of magnitude lower than AEG and DAB. Seasonal measurements conducted at two bloom-impacted lakes indicated limited correlations of BMAA isomers with total microcystins or chlorophyll-a, which deserves further investigation.

## 1. Introduction

Cyanobacteria produce a host of secondary metabolites, some of which can present toxic effects in plants, invertebrates, and vertebrates during acute or chronic exposures [1]. The neurotoxin β-N-methyl-amino-L-alanine (BMAA) is a nonproteinogenic amino acid suspected to be implicated in the etiology of neurodegenerative diseases [2,3,4,5,6,7,8]. It was originally discovered in the Pacific Island of Guam, where it was produced by the cyanobacterium *Nostoc* sp. in endosymbiosis within the coralloid roots of cycads [9]. BMAA exposure (through the consumption of cycad seed flour and flying fox bats that bioconcentrated the toxin) was suggested as one of the possible factors for the high incidence rates of amyotrophic lateral sclerosis and endemic neuronal diseases observed there. BMAA and some of its structural isomers, including N-(2-aminoethyl) glycine (AEG), β-amino-N-methylalanine (BAMA), and 2,4-diaminobutyric acid (DAB), may also be produced in aquatic ecosystems during cyanobacterial harmful algal blooms (CyanoHABs). Phytoplanktonic phyla other than cyanobacteria, including ochrophyta (photosynthetic heterokonts, e.g., diatoms) and myzozoa (e.g., dinoflagellates), may also be capable of biosynthesis [10]. Hence, accidental ingestion of contaminated surface water during recreational activities and the consumption of contaminated drinking water, fish, shellfish [11], and spirulina food supplements [12] are other potential human exposure routes.

BMAA typically occurs at low–moderate levels in free-living cyanobacteria (ng g^−1^ to µg g^−1^ dry weight) [10,13,14,15,16] and presumably at much lower levels in bulk surface waters (extracellular dissolved, ng L^−1^ to µg L^−1^) [15,17,18,19]. This implies that BMAA and its isomers may escape detection in environmental waters. In addition, few studies have targeted environmental waters for BMAA monitoring, compared with biological samples [10].

A trace analysis of BMAA can be complex to implement, as described in several reviews [13,20,21]. The re-evaluation of pre-existing analytical methods has also demonstrated the potential for QA/QC failures [22,23]. Difficult chromatographic retention of underivatized BMAA and its resolution from co-occurring isomers (e.g., AEG, BAMA, and DAB) are well-known issues, but there exist other pitfalls. For instance, Roy-Lachapelle et al. [19] showed that dissolved AEG, BMAA, and DAB could suffer significant losses (>60%) with certain types of filtration materials, including glass fiber filters (GFF). As a basic amino acid, BMAA could also adhere to the surface of other glassware materials [21], potentially resulting in underreporting of concentrations. There are also cases where overreporting may be observed. Faassen et al. [22] evaluated a commercial enzyme linked immunosorbent assay (ELISA) and showed that the kit was not suitable for BMAA screening in surface waters. Recoveries were as high as 400% in spiked samples, and the ELISA kit yielded positive BMAA results in nearly all field samples (max of 300 µg L^−1^ for pond water), contrasting with no detections in the corresponding split samples submitted to LC-MS/MS [22]. A similar issue was recently noted by Zhang et al. [24] during the analysis of BMAA in surface runoff waters by the two instrumental techniques. The false positive results sometimes obtained with ELISA in natural waters [22,24] and other complex matrixes [25] were likely due to interferences with co-occurring matrix components. A critical review of the BMAA literature also noted serious flaws in the validation and reporting of analytical procedures [13]. The use of unsuited or insufficiently documented analytical methods may partly explain the BMAA controversy, i.e., the current lack of consensus over the widespread presence of BMAA in aquatic ecosystems [13,21].

Another critical aspect relates to the fact that BMAA can exist in different forms. For instance, for solid samples, free, soluble bound, and precipitated bound BMAA fractions have been investigated [26]. Faassen et al. confirmed that solid biological samples (cycad seeds, seafood, and exposed cladocerans) pre-treated with trichloroacetic acid (TCA) and hydrochloric acid (HCl) had BMAA concentrations greatly surpassing the free BMAA fraction [26]. Lage et al. found higher recoveries of BMAA from Spirulina powder (*Anthrospira fusiformis*) with an aqueous solution of 0.1 M TCA, compared with solvent mixtures without TCA (e.g., methanol/water or methanol/acetone) [27]. In the previous workflows targeting BMAA in surface water, the samples typically were not amended with strong acids [15,19,28,29]. Although SPE (on-line or off-line) was used as a pre-concentration step, BMAA was rarely detected in lake water [19,29]. BMAA isomers might be bound with metals, organic colloids, or cell residues in water, and this may lead to underestimation using the currently available procedures for surface water samples. Whether amending the lake samples with strong acid would yield different results has not been previously explored.

**Figure 1:**
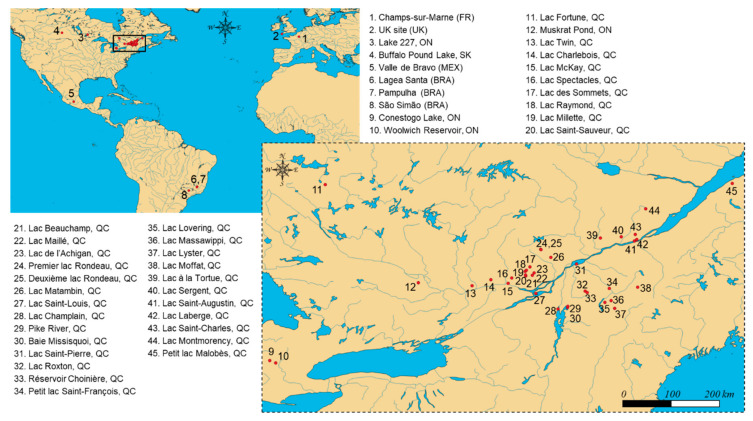
Geographical location of the 45 sampling sites monitored in the present survey, including lakes and reservoirs of Brazil (BRA), Canada (Ontario (ON), Québec (QC), and Saskatchewan (SK)), France (FR), Mexico (MEX), and the United Kingdom (UK). Sampling was conducted at different locations within some lakes to account for local variations of CyanoHABs (e.g., sites #4, #5, #9, #28, #30, and #34), different water column depths (e.g., site #4), or different days during seasonal high-intensity sampling (e.g., sites #4 and #34), leading to an overall number of *n* = 390 environmental water samples for analysis.

The large number of lakes targeted for cyanotoxin analysis within the framework of the ATRAPP project (Algal Blooms, Treatment, Risk Assessment, Prediction, and Prevention) provided an opportunity to address the knowledge gap. We also aimed to confirm whether BMAA and its isomers would be widely occurring in freshwater lakes impacted by harmful cyanobacterial blooms. In the first step, field-collected surface water samples were processed by two different methods to evaluate the influence of 0.1 M TCA amendment (method A: without TCA; method B: with TCA). A sensitive instrumental method relying on the on-line enrichment of derivatized BMAA/isomers and liquid chromatography high-resolution mass spectrometry was applied [29]. In the second step, we used method B (with TCA) to examine the presence of AEG, BAMA, BMAA, and DAB in a larger number of environmental water samples. The monitoring included 390 samples from 45 lakes and reservoirs of Brazil, Canada, France, Mexico, and the United Kingdom (Figure 1). To the best of our knowledge, this is the first study to report on the occurrence of BMAA isomers in lake samples at such a large spatial scale.

## 2. Results and Discussion

### 2.1. Influence of TCA Addition

Surface water samples were analyzed using two sample preparation methods: A: without TCA amendment [19,29] and B: with 0.1 M TCA [26,30,31].

Of 158 surface water samples analyzed with both methods (Supplementary Material Table S3), 69 samples (43%) were positive for at least one of the four isomers with method A, while higher detection rates were obtained with the addition of 0.1 M TCA (119/158 positive samples or 75%). Compound-dependent detection rates were also lower using method A (AEG 19%, BAMA 3%, and DAB 35%) than method B (AEG 42%, BAMA 15%, and DAB 62%). Interestingly, BMAA itself was not detected in any of the surface water samples using either method (Supplementary Material Table S3), contrasting with the relatively widespread occurrence of its isomers AEG and DAB. In a previous study [29], we detected BMAA in only one sample (out of 82) using the same analytical approach as method A.

**Figure 2:**
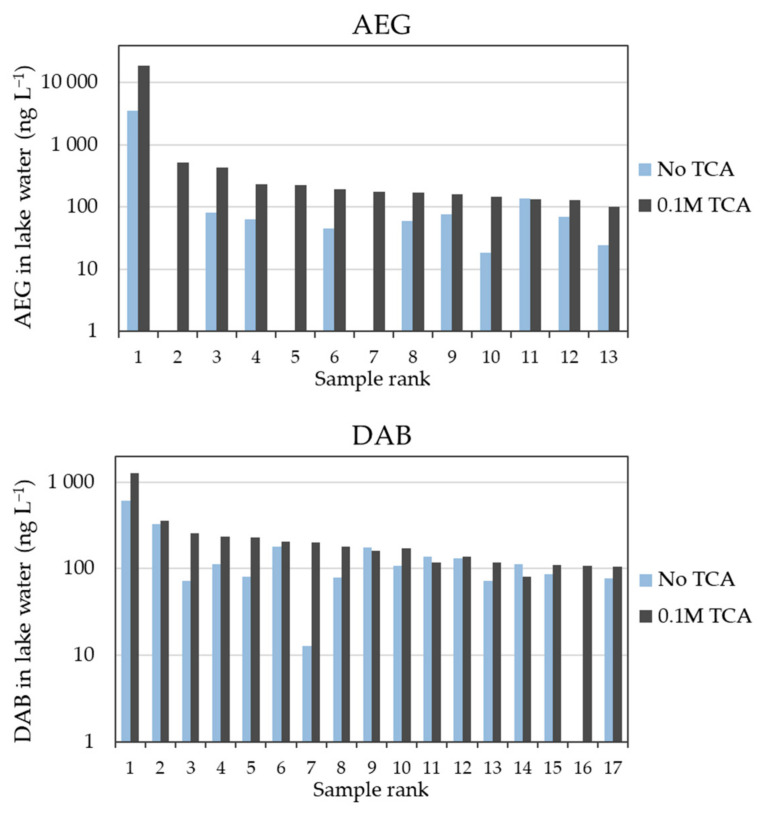
Quantified concentrations (ng L^−1^) of AEG and DAB in surface water samples with or without 0.1 M TCA, for those samples with concentrations greater than 100 ng L^−1^ (in the figure *x*-axis, samples are ranked according to decreasing AEG and DAB concentrations by either method). Due to the ranges of concentrations in environmental samples from different locations, a logarithmic scale was applied to the *y*-axis. The AEG data below the detection limit of 5 ng L^−1^ for three samples (rank: 2, 5, and 7) without TCA added; DAB data below the detection limit of 10 ng L^−1^ for one sample (rank: 16) without TCA added.

The concentrations of BMAA isomers were significantly greater using method B vs. method A (paired Wilcoxon’s signed-rank tests, *p*-values: *p*_AEG_ and *p*_DAB_ < 0.001). When a concentration value could be simultaneously returned by both methods (matching pairs calculation method), concentration ratios (B/A) greater than 1 were frequently observed. Samples with concentrations greater than 100 ng L^−1^ (by either method) almost systematically followed the B > A trend (Figure 2), with median ratios of 3.9 (i.e., +290% with TCA amendment) and 1.5 (+50%) for AEG and DAB, respectively. At the site location with the highest AEG concentration (Petit lac Saint-François), amending the sample with TCA led to an increase of about 5.3-times (+430%) the determined concentration (19,000 vs. 3600 ng L^−1^).

Median ratios higher than their unity were also observed for the full scope of samples without concentration thresholds (i.e., median B/A of 2.4 and 1.3 for AEG and DAB, respectively). The overall range of B/A ratios were 0.67–8.25 for AEG (i.e., up to +725% with TCA) and 0.69–15.5 for DAB (i.e., up to +1450% with TCA). While a slightly negative influence (i.e., down to −33%) could be considered to fall within the analytical variation, the large increase observed at several locations could not. In addition, the use of internal standardization (BMAA-d3 spiked before derivatization) and matrix-matched calibration for each protocol likely corrected for possible instrumental variations [29].

Based on the above, we can conclude that TCA addition had a significant influence on detection rates and concentrations of BMAA isomers in lake water. To the best of our knowledge, this is the first study to report on this type of impact for water samples. The complexation of amino acids with metallic or organic ligands may be reduced under the low pH conditions of method B (pH < 1) [32] compared with method A (circumneutral pH). Low pH conditions could also favor the precipitation or denaturation of medium- and long-chain peptides [33] (e.g., originated from cellular debris) that could otherwise engage in hydrogen bond interactions. This could explain the different detection trends of BMAA isomers between the two methods. The magnitude of the increase with the TCA method was also slightly higher for samples with greater concentrations of BMAA isomers, which could reflect the enhanced sequestration potential in the more complex water bloom samples unless treated with method B. The addition of TCA may be recommended for future studies targeting dissolved BMAA in ambient water, while stronger conditions (e.g., involving additional hydrolysis with 6 M HCl) would be required for biotic tissues and lyophilized cyanobacterial samples [26].

### 2.2. Occurrence Trends of BMAA Isomers in 390 Lake Samples

Overall, 390 surface water samples from 45 different locations (Figure 1) were analyzed for BMAA isomers, using the 0.1 M TCA method. The associated descriptive statistics, including detection rates and concentration ranges, are summarized in Table 1. Detailed concentrations per sample and aggregated literature data from an additional 136 samples (Table 2) are also provided in the Supporting Excel data.

Of 390 samples, 222 (57%) were positive to at least one of the BMAA isomers. AEG and DAB were the most recurrently detected isomers (30% and 43% of samples with hits), while BAMA was found in only 7.4% of samples and BMAA was not found above the method detection limit (LOD of 10 ng L^−1^) in any sample. AEG and DAB co-occurred in 16% of the samples, while 6% of the samples had detectable levels for the three isomers simultaneously (AEG, BAMA, and DAB). The number of lakes with positive detections was 21/45 for AEG and 15/45 for DAB. BAMA was found at five site locations only (Buffalo Pound Lake, Conestogo Lake, Lac Saint-Augustin, Petit lac Saint-François, and Woolwich Reservoir).

Ten locations presented maximum concentrations of AEG and/or DAB above 100 ng L^−1^: Buffalo Pound Lake, Conestogo Lake, Lac Beauchamp, Lac Fortune, Lac Millette, Lac Saint-Augustin, Lac Saint-Pierre, Missisquoi Bay, Petit lac Saint-François, and the UK site. The maximum observed concentrations for each isomer were 19,000 ng L^−1^ for AEG (Petit lac Saint-François), 1100 ng L^−1^ for DAB (Petit lac Saint-François), and 56 ng L^−1^ for BAMA (Buffalo Pound Lake) (Table 1). The maximum concentrations of BMAA isomers in the μg L^−1^ range agreed with previous surveys of CyanoHAB impacted lakes and reservoirs in Canada [17,29] and the United States [18] (Table 2). Detailed concentrations per sample and aggregated literature data from an additional 136 samples (Table 2) are also provided in the Supporting Excel data.

Relatively high concentrations of AEG and DAB in some samples and the concomitant lack of BMAA detection agreed with a previous survey of lakes and rivers from Eastern Canada [29]. The results could also be related to a monitoring survey of cyanobacterial blooms in Lake Winnipeg, Manitoba, Canada [16]. In that study, the levels of AEG and DAB found in cyanobacterial samples (averages of 2120 and 170 μg g^−1^) were 2–3 orders of magnitude higher than those of BMAA (average of 4.05 μg g^−1^) [16]. A similar trend was observed in marine mats of the Arabian Gulf (Khor Al Adaid) and Australian cyanobacterial blooms, with AEG and/or DAB often 3–4 orders of magnitude higher than BMAA [15,34]. If BMAA was present in surface water from our survey, concentrations 2–4 orders of magnitude lower than AEG or DAB would fall below the detection threshold (10 ng L^−1^). While a few studies did report detectable levels of BMAA in lake water samples [18,19] (see also Table 2), the present study and other surveys [15,16,29,34] suggest that AEG and DAB isomers may be more prevalent than BMAA. However, monitoring of a much higher number of aquatic ecosystems would be needed for confirmation.

### 2.3. Monitoring of Two CyanoHAB Impacted Lakes during the Bloom Season

Monitoring conducted at two sites allowed us to examine time trends of BMAA isomers during the bloom season (Buffalo Pound Lake; BPL) or throughout the entire year (Petit lac Saint-François; PLSF, also referred to as Lake Tomcod in the literature). The selection of sampling sites for this study was aligned with previous reports of relatively high AEG/DAB levels at these two locations, compared with a range of other Canadian lakes from New Brunswick, Nova Scotia, Ontario, Québec, and Saskatchewan [16,19,29]. Both BPL and PLSF have also been experiencing significant cyanobacterial bloom-related stressors [35,36].

**Figure 3:**
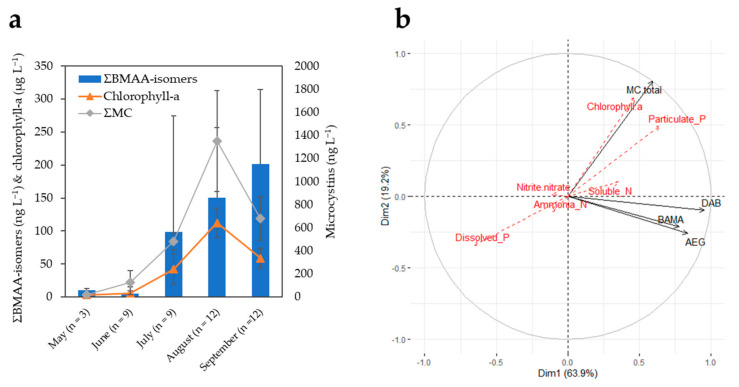
(**a**) Monthly averages of summed BMAA isomers (ng L^−1^), chlorophyll-a (μg L^−1^), and total microcystins (ng L^−1^) (MMPB method) in Buffalo Pound Lake during the 2019 sampling season. Error bars are the corresponding standard deviations. Summed BMAA isomers and chlorophyll-a are plotted against the primary (left) y-axis, while the secondary (right) y-axis is used for total microcystins. (The different y-axis scales are for visualization purposes only, and they do not refer to scaled toxicities). (**b**) Principal component analysis (PCA) correlation circle of active variables (total MCs, AEG, BAMA, and DAB) and additional environmental variables superimposed on the plot (red font dotted arrows). To deal with different units/scales between environmental variables, data were transformed (center-reduced) prior to performing the PCA. Temperature data were not available for all dates and, therefore, could not be included in the PCA.

BPL is a shallow polymictic lake of an elongated shape (~29 km × 1 km, max. depth of 5.8 m) located in the Canadian prairies. It also serves as a major drinking water supply for the cities of Moose Jaw and Regina [37], representing 25% of Saskatchewan’s population. Occurrences of harmful cyanobacterial algal blooms in BPL and the related poisoning of dogs, cattle, and poultry were reported as early as the 1960s [37]. The BPL campaign included 49 water samples collected between 29 May and 3 October 2019, examined for BMAA isomers and other parameters (Figure 3).

The monthly concentrations of summed BMAA isomers (ΣBMAA-isomers: AEG + BAMA + DAB) in BPL water samples are plotted in Figure 3a. The average concentrations of BMAA isomers remained at or below detection limits in the late spring months (~6–10 ng L^−1^), increased by about 10–20 times in July–August (~100–150 ng L^−1^) concomitantly with total microcystins (ΣMC), and were still increasing in September (~200 ng L^−1^), while the levels of ΣMC had already receded. In Figure 3b, vectors of AEG, BAMA, and DAB are on the same PCA correlation circle quadrant (i.e., highly correlated together) but near-orthogonal (i.e., unrelated) to those of ΣMC and chlorophyll-a. Pip et al. also observed nonsignificant or weakly significant correlations of BMAA and microcystins in water samples of Lake Winnipeg, Canada [17]. BMAA and isomers may be produced by later-blooming communities compared with those responsible for the observed peak of ΣMC. The related BPL taxonomic identification suggested a shift in community composition from *Dolichospermum flos aquae* to *Planktothrix agardhii* dominated blooms during the 2019 season, which might be associated with different toxigenic cyanopeptide profiles, as recently discussed in Painter et al. [38].

**Figure 4:**
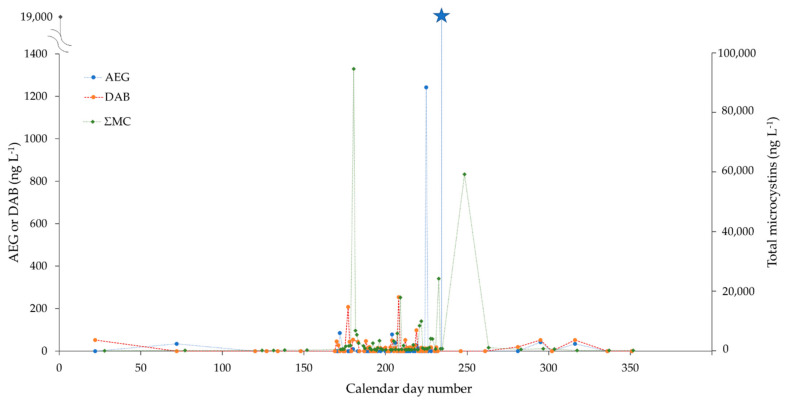
Concentrations of AEG (ng L^−1^), DAB (ng L^−1^) and total microcystins (ng L^−1^) (MMPB method) in lake water samples of Petit lac Saint-François, collected between January and December 2019 (*x*-axis: calendar day number). High-frequency intensity sampling was conducted during the two-month summer bloom period (June–August), with near-daily sample collections. AEG and DAB concentrations are shown on the primary (left) *y*-axis, while total microcystins are plotted against the secondary (right) *y*-axis. (The different *y*-axis scales are for visualization purposes only, and they do not refer to scaled toxicities). The primary axis break was applied to plot an extremely high value of AEG observed on 20 August 2019 (19,000 ng L^−1^, blue star marker). For each time point and measured parameter, only a single datum point was provided; hence, error bars cannot be displayed.

PLSF is a shallow hypereutrophic lake (1.7 km × 0.7 km, max. depth of 2 m) located in the municipality of Saint-François-Xavier-de-Brompton (Estrie, QC, Canada). Nutrient physicochemistry indicates a relatively poor water quality status and advanced eutrophication [39,40]. A subset of 66 PLSF water samples collected between 22 January and 17 December 2019 were included for analysis (Figure 4). A high sampling intensity was achieved during the two-month summer bloom period, with near-daily sample collections. Concentrations of AEG and DAB were low in the winter and spring seasons (<5–50 ng L^−1^), at or near the background levels of Québec rivers without known CyanoHAB impacts ([29]; see also Supporting Excel data). The AEG concentrations drastically increased at ca. 1200 ng L^−1^ in mid-August and then peaked to even higher levels (19,000 ng L^−1^) one week later, seemingly unrelated to ΣMC (Figure 4). Even after removing these two outlier points, the vector of AEG remained orthogonal to that of ΣMC (and DAB) on the PCA correlation circle (Supplementary Material Figure S2). The early summer bloom was dominated by *Aphanizomenon flos-aquae*, *Dolichospermum* spp., and *Microcystis aeruginosa* (June–July 2019); similar to BPL, the contribution of *Planktothrix agardhii* increased during the later part of the summer for PLSF. However, biomass is not *per se* indicative of toxin production (microcystis biomass and microcystins do, in general, show a correlation, but this does not always occur with other toxins or other cyanobacteria species). Although clearly beyond the scope of the present study, multi-year seasonal monitoring and acquisition of metagenome sequencing data may help clarify these trends.

## 3. Conclusions

In this study, we evaluated the trends of BMAA isomers in lake water with two protocol variations. We observed that previously published procedures that did not involve TCA addition may have led to lower levels of extracellular concentrations of BMAA isomers, compared with TCA-amended samples. The magnitude of the difference was variable depending on the water source but could reach values as high as 15-times for the TCA method (and a maximum observed magnitude difference of +15,000 ng/L for AEG). The standard TCA concentration used in our study was derived from the literature on BMAA analysis of solids, such as cycad seeds, shellfish, and powdered cyanobacteria [26,27]. The herein described procedure may still require additional refinement, including optimization of the TCA concentration [27] or investigation of hydrolysis under stronger conditions, such as concentrated HCl/heat [26]. Since our current method involved derivatization and a large volume injection (1 mL) by on-line SPE-UHPLC-HRMS, it was not deemed feasible to include a 6 M HCl hydrolysis step of the bulk lake water. To avoid substantial sample dilution, testing of a harsher hydrolysis step would likely necessitate pre-emptive freeze-drying of the lake water aliquots prior to reconstitution in a small volume of concentrated HCl.

The TCA method was applied to a large set of field-collected lake samples from America and Europe (overall *n* = 390). BMAA was not found (LOD of 10 ng L^−1^) in any of the 45 lakes monitored during the bloom season, while two of its isomers, AEG and DAB, could reach peak concentrations in the μg L^−1^ range. The results from the present study and a few others [15,16,29,34,41] indicate that BMAA may be less common in freshwater cyanobacterial algal blooms than previously thought. The most frequent occurrences of AEG and DAB were reported here for lakes with known cyanobacterial bloom impacts and sampled during the summer–fall bloom season. Much lower detection rates were observed here in the winter–spring season for the two lakes submitted to temporal monitoring. BMAA isomers are also expected to fall in the low or nondetectable ranges for other types of water bodies, including rivers [29].

While DAB may show similar neurotoxic effects as BMAA [42], in vitro bioassays suggest that AEG may be less toxic than BMAA and DAB [43,44]. Although no guidelines are currently available for BMAA in water, we note that peak levels of total microcystins far exceeded the interim drinking water advisory levels in one of the lakes targeted for seasonal monitoring. With some modifications of the sample preparation or instrumental procedures, the sensitive method could be expanded to the study of BMAA isomers in fish, shellfish, and spirulina food supplements in future work. Future surveillance and cyanotoxin risk assessment studies in bloom-impacted freshwater and marine ecosystems should preferably include analysis of BMAA isomers along with other cyanotoxins and taxonomic analysis of the phytoplankton.

## 4. Materials and Methods

### 4.1. Chemicals and Standards

N-(2-aminoethyl) glycine (AEG) was obtained from Toronto Research Chemicals Inc. (North York, ON, Canada). β-N-methylamino-L-alanine hydrochloride (L-BMAA) (purity ≥ 97.0%) and L-2-2-diaminobutyric acid dihydrochloride (DAB) (purity ≥ 95.0%) were purchased from Sigma Aldrich (Oakville, ON, Canada). β-amino-N-methyl-alanine (BAMA) was acquired from the National Research Council of Canada (Halifax, NS, Canada). The isotope-labelled internal standard L-BMAA hydrochloride-d3 (BMAA-d3) was purchased from Abraxis, Inc. (Warminster, PA, USA).

Acetonitrile (ACN), methanol (MeOH), and water of HPLC quality were purchased from Fisher Scientific (Whitby, ON, Canada). Ammonium acetate (purity ≥ 98%), sodium citrate dibasic sesquihydrate (citrate; purity 99.0%), sodium tetraborate decahydrate (borate; purity 99.5%), potassium hydroxide (KOH; purity 90%), and trichloroacetic acid (TCA; purity ≥ 99.0%) were purchased from Sigma Aldrich (Oakville, ON, Canada). The derivatizing agent 9-fluorenylmethyl chloroformate (FMOC-Cl; 98.0% purity) was obtained from Alfa AeSar (Fisher Scientific, Whitby, ON, Canada).

### 4.2. Sample Collections

Surface water samples (*n* = 390) were collected in 2016–2021 from 45 locations in Brazil, Canada (from the provinces of Ontario, Québec, and Saskatchewan), France, Mexico, and the United Kingdom. Their geographical distribution is illustrated in Figure 1. Several of these sites corresponded to freshwater lakes with a documented history of harmful cyanobacterial blooms [38,39,40,45,46,47]. Sampling was conducted as part of the ATRAPP project by trained university staff and partners. Additional water samples from bloom-impacted lakes were obtained through a citizen-science project (Adopt a Lake) [48]. Surface water samples for cyanotoxin analysis were collected in 125-mL amber polyethylene terephthalate glycol-modified (PETG) bottles, previously washed in the laboratory and rinsed in situ three times with the site surface water. Samples were kept at 4 °C and shipped within 1–3 days of collection to the analytical facilities, where they were lysed (three freeze–thaw cycles) prior to storage at −20 °C [49] until preparation and analysis.

In addition, 500-mL wide-mouth high-density polyethylene (HDPE) bottles (opaque, acid-washed) were co-collected for the analysis of the ancillary surface water parameters, such as nutrients and chlorophyll-a; filtered; and stored at 4 °C until analysis. Nutrient chemistry analyses were performed at the University of Saskatchewan Global Institute for Water Security (Saskatoon, SK, Canada), Environmental Geochemistry Laboratory University of Waterloo (Waterloo, ON, Canada) and Université de Montréal Department of Biological Sciences (Montreal, QC, Canada) using standard methods. Taxonomic analyses were conducted at Water’s Edge Scientific LLC (Baraboo, WI, USA). Preserved samples were analyzed according to APHA Method 10200F (APHA, 2012), and cell counting was performed in a Sedgewick-Rafter counting chamber.

### 4.3. Sample Preparation

The analysis of surface water samples by method B (with TCA) was performed as follows. An aliquot of the lysed surface water was filtered through a nitrocellulose filter (0.2 μm, 25 mm) fitted onto a syringe filter holder. A five-mL aliquot of the filtrate was then spiked with the isotopically-labelled internal standard (ILIS: BMAA-d3) to achieve an initial concentration of 500 ng L^−1^. TCA was added to achieve a concentration of 0.1 M [26,30], followed by a 10-min wait time. The solution was brought to a circumneutral pH with KOH prior to the addition of buffer solutions of borate (0.3 mL of a 100 mM solution) and citrate (0.3 mL of a 150 mM solution) to achieve a pH of ~9. The mixture was vortexed for 10 s (3200 rpm), followed by a five-min wait time. FMOC-Cl was added to the samples (300 μL of a 3 mg mL^−1^ solution prepared in ACN), and the derivatization reaction proceeded for 1 h while stirring (200 rpm, lab oven shaker; 65 °C; without light). The samples were left to cool to room temperature, and an organic cosolvent (300 µL of MeOH) was subsequently added to quench the reaction and minimize sorption losses of FMOC-derived amino acids [29]. Finally, reacted samples were vortexed (10 s; 3200 rpm) and centrifuged (10 min; 6000 rpm) prior to aliquoting of 1.5 mL of the supernatant in a two-mL LC-MS vial.

A subset of the surface water samples was also analyzed by method A (without TCA added) [29]. Lysed water samples were filtered (nitrocellulose, 0.2 μm, 25 mm), spiked with BMAA-D3 (500 ng L^−1^), amended with borate and citrate buffers, and derivatized with FMOC-Cl as previously described.

### 4.4. Instrumental Analysis

Derivatized samples were analyzed by on-line solid-phase extraction (on-line SPE) coupled with ultra-high-performance liquid chromatography high-resolution mass spectrometry (UHPLC-HRMS), adapted from Vo Duy et al. [29] with some modifications.

An injection was performed using a PAL RTC autosampler (Zwingen, Switzerland) and a one-mL stainless-steel loop (SST). The injection volume was set at 1 mL. On-line enrichment was performed with a Thermo Dionex UltiMate™ 3400 SD pump and a Thermo HyperSep Retain PEP column (hydrophilic lipophilic balance, 20 mm × 2.1 mm, particle size 40–60 μm). The loading flow rate was set at 1500 μL min^−1^. After sample loading, the on-line aqueous mobile phase (HPLC water) was allowed to flow for an additional 2 mL to remove salts.

The target analytes were then eluted at 450 μL min ^−1^ in back-flush mode with the analytical mobile phase (A: 2.5 mM CH_3_COONH_4_ in HPLC water; B: ACN), using a Thermo Dionex UltiMate™ 3400 RS pump. For UHPLC separation, a Thermo Hypersil Gold C18 column (100 mm × 2.1 mm, particle size 1.9 µm, pore size 175 Å) thermostated at 35 °C was used. The UHPLC column was fitted with a 0.2-µm column prefilter. Details on the chromatographic elution gradient are provided in Supplementary Material (Table S1), as are representative UHPLC-HRMS chromatograms showing the separation of the four isomers in spiked lake water (Supplementary Material Figure S1).

Analyte detection was performed using negative electrospray ionization (ESI) and a Thermo Q-Exactive Orbitrap mass spectrometer (Thermo Scientific, San Jose, CA, USA), with a full scan MS range of *m*/*z* 200–600 and a resolution setting of 70,000 full width at half maximum (FWHM; value at *m*/*z* 200). Further details on the ESI source and MS acquisition parameters are provided in Supplementary Material (Table S1).

Total microcystins (ΣMC) were examined in conjunction with BMAA/isomers for the high-intensity sampling sites (Figure 1, sites #4 and #34). The ΣMC were analyzed in the nitrocellulose-filtered fraction of the lysed water sample. The 2-methyl-3-methoxy-4-phenylbutyric acid (MMPB) moiety generated via Lemieux-von Rudloff oxidation was analyzed using a previously validated method [50]. An aliquot of the reacted sample was spiked with ILIS (MMPB-d3) and analyzed by on-line SPE coupled to UHPLC tandem mass spectrometry (TSQ Quantiva LC-MS/MS, Thermo Scientific, San Jose, CA, USA). Further details on the analytical method for ΣMC are summarized in Supplementary Material (Text S1 and Table S2).

### 4.5. Quality Assurance/Quality Control (QA/QC)

The identification of BMAA and its isomers in surface water samples was based on matching retention times (±0.1 min) with calibration curve standards (e.g., Supplementary Material Figure S1) and mass accuracy of observed vs. theoretical exact *m*/*z* (tolerance of ±5 ppm).

The analytical method without TCA (method A) was previously subject to matrix-matched validation in lake water [29], including the assessment of linearity (R^2^ = 0.9963–0.9982), whole-method accuracy (spike level of 75 ng L^−1^; accuracy = 76–101%), and intermediate precision (75 ng L^−1^; intraday RSD of 2.1–6.7% and interday RSD of 8.1–13%). Matrix-matched calibration was adopted, and relative matrix effects (standard additions to select lake water samples) were within −22% to +17% [29].

Following the initial demonstration of method capability [29], continued QA/QC measures were implemented for the present survey, for both methods A and B. Method blanks were performed for each batch of samples using surface water aliquots from Lac Pohénégamook (QC, Canada) and submitted to the entire preparation procedure; no contamination was noted. Method detection limits in the present study were 5 ng L^−1^ for AEG and 10 ng L^−1^ for BAMA, BMAA, and DAB. An eight-point based matrix-matched calibration curve (15–1000 ng L^−1^, additions to a blank lake matrix from Lac Pohénégamook, QC, Canada, subsequently submitted to either method A or B) was performed at the beginning of each LC-MS sequence. Determination coefficients (R^2^) were within the typical range of 0.995–0.999. After the initial calibration, continued calibration verification (CCV) standards were run as matrix spikes (fortification level: 75 ng L^−1^). The accuracy of CCV standards was required to fall within 70–120% [51].

### 4.6. Data Curation and Statistical Analyses

Processing of LC-MS data was performed using the Xcalibur 4.3 software (Thermo Scientific). Statistical analyses were conducted with the R statistical software version 4.1.1 (R Core Team [52]). Statistical significance was set at *p* <0.05. A Principal Component Analysis (PCA) of center-reduced data was performed using the *FactoMineR* R-package (graphs plotted with *factoextra* and *ggplot2*). Wilcoxon’s signed rank tests for paired data were used to evaluate the statistical differences between TCA and non-TCA treatments; statistical differences could be investigated for AEG and DAB only, i.e., the two compounds with sufficient detections with both treatments. The map of sample locations was designed using Quantum GIS (*QGIS 3.6 Noosa*) as a geographic information system, and the base maps were obtained from Natural Earth (free vector and raster map data available at naturalearthdata.com, accessed on 6 January 2022).

## Figures and Tables

**Table 1:** Descriptive statistics, including detection frequency (% of samples >LOD) and concentration ranges (min–max and average, ng L^−1^) of BMAA isomers in surface water samples (*n* = 390, TCA method). Site locations with concentrations punctually surpassing 100 ng L^−1^ are also indicated (maximum observed concentration parenthetically noted). PLSF: Petit lac Saint-Francois; BPL: Buffalo Pound Lake.

	AEG	BAMA	BMAA	DAB
LOD (ng L^−1^)	5	10	10	10
Detection rate %	30	7.4	0	43
Min (ng L^−1^)	5	15	-	10
Max (ng L^−1^)	19,000	56	-	1100
Average * (ng L^−1^)	220	NC ***	NC ***	58
Average ** (ng L^−1^)	67	NC ***	NC ***	25
Sites >100 ng L^−1^ (max, ng L^−1^)	PLSF (19,000) BPL (518) Conestogo (225) Lac Millette (178) UK site (125)	-	-	PLSF (1100) Lac Saint-Augustin (359) Lac Fortune (316) Lac Millette (230) Lac Saint-Pierre (224) Lac Beauchamp (136) BPL (119) Missisquoi Bay (109)

* Average of samples with positive detections only. ** Average of all samples (*n* = 390) including non-detect data. *** NC: Not calculated. The average was not calculated for compounds of low detection frequencies.

**Table 2:** Overview of literature data reporting BMAA and its isomers in water samples of freshwater (lakes, rivers, and reservoirs) and saltwater (seawater) environments, including instrumental methods, limits of detection (LOD, ng L^−1^), type and number of field-collected samples, and concentration ranges (min-max of positive samples, ng L^−1^). Sample-specific concentration data of the present study and aggregated literature are also provided in the Supporting Excel data.

	Abbes et al. (This Study)	Al Samaak et al. [18]	Chatziefthimiou et al. [15]	Roy-Lachapelle et al. [19]	Vo Duy et al. [29]
Instrumental method	LC-HRMS	LC-FLD	LC-MS	LC-HRMS	LC-HRMS
LOD range ng L^−1^	5–10	5000–7000	N/A	7–9	2–5
Water matrix	Lakes	Lakes	Seawater	Lakes	Lakes & rivers
Site location(s)	5 countries *	USA	Qatar	Canada	Canada
Year of collections	2016–2021	2009–2010	2012–2013	2009, 2013	2016–2018
Number of samples	*n* = 390	*n* = 24	*n* = 18	*n* = 12	*n* = 82
AEG (min-max) ng L^−1^	5–19,000	Not analyzed	35/38 **	9–80	2–4900
BAMA (min-max) ng L^−1^	15–56	Not analyzed	Not analyzed	Not analyzed	41–130
BMAA (min-max) ng L^−1^	Not detected	1800–25,300	6.5/7 **	10–300	110 ***
DAB (min-max) ng L^−1^	10–1100	1780–21,100	430/610 **	8–40	13–1900

* Brazil, Canada, France, Mexico, and the United Kingdom. ** Mean of positive samples from the 2012/2013 surveys. *** Only one sample above the LOD.

## Data Availability

Data available as a Supporting Information Excel file.

## Supplementary Materials

The following supporting information can be downloaded at: https://www.mdpi.com/article/10.3390/toxins14040251/s1, Text S1: Analysis of total microcystins via Lemieux-von Rudloff oxidation, Figure S1: UHPLC-HRMS chromatograms of FMOC-derivatized BMAA isomers (AEG, BAMA, BMAA, and DAB) spiked at 50 ng L^−1^ in blank lake water. The lower pane shows the corresponding isotope-labeled internal standard (ILIS: BMAA-D3) spiked at 500 ng L^−1^, Figure S2: Principal component analysis (PCA) correlation circle of active variables (total MCs, AEG, DAB) and additional environmental variables superimposed on the plot (red font dotted arrows) for the PLSF site. The PCA is applied to a subset of *n* = 64 samples (two outliers removed). Chlorophyll-a data were not available for all time points and therefore could not be included in the statistical analysis, Table S1: Details on the UHPLC-HRMS instrumental method for the analysis of FMOC-derivatized BMAA and its isomers, Table S2: Details on the UHPLC-MS/MS instrumental method for the analysis of total microcystins via oxidative cleavage (MMPB method), Table S3: Measured concentrations (ng L^−1^) of AEG, BAMA, BMAA, and DAB in samples of freshwater lakes and reservoirs, with or without amendment of 0.1M TCA. Sample-specific concentration data of the present study and aggregated literature are also provided in the Supporting Excel data.
