# Supplementary material for: Occurrence of BMAA Isomers in Bloom-Impacted Lakes and Reservoirs of Brazil, Canada, France, Mexico, and the United Kingdom
Source: Toxins (Basel). 2022 Mar 31;14(4):251. doi: 10.3390/toxins14040251 (PMC9026818; doi:10.3390/toxins14040251)
Supplement: Supplementary file 1 [file toxins-14-00251-s001.zip › toxins-1621664-supplementary.pdf]

Supplementary Materials: Occurrence of BMAA Isomers in Bloom-Impacted Lakes and Reservoirs of Brazil, Canada, France, Mexico, and the United Kingdom

Safa Abbes, Sung Vo Duy, Gabriel Munoz, Quoc Tuc Dinh, Dana F. Simon, Barry Husk, Helen M. Baulch, Brigitte Vinçon-Leite, Nathalie Fortin, Charles W. Greer, Megan L. Larsen, Jason J. Venkiteswaran, Felipe Fernando Martínez Jerónimo, Alessandra Giani, Chris D. Lowe, Nicolas Tromas and Sébastien Sauvé

Text S1. Analysis of total microcystins via Lemieux-von Rudloff oxidation.

Surface water samples from two high-intensity monitoring sites were also analyzed for total microcystins (ΣMC). The method was based on Lemieux-von Rudloff oxidation,[1] which generates 2-methyl-3-methoxy-4-phenylbutyric acid (MMPB) upon cleavage of the ADDA moiety common to most microcystins and nodularins. We used a previously optimized and validated method,[2] with minor modifications. Environmental water samples (freshwater lakes and reservoirs) were amended with potassium permanganate and sodium periodate (50 mM each) and adjusted to pH 9 using potassium carbonate. The oxidation reaction proceeded for 60 min under magnetic stirring, after which samples were quenched with sodium bisulfite. Samples were amended with the isotope-labeled internal standard (ILIS: D3-MMPB, 100 ng L^{-1}) and passed through nylon filters (0.22 μm). A 1-mL aliquot of the oxidized samples was analyzed by on-line solid-phase extraction (on-line SPE) coupled to ultra-high-performance liquid chromatography tandem mass spectrometry (Thermo TSQ Quantiva UHPLC-MS/MS). Details on the applied chromatographic gradient programs and other instrumental settings are provided in SI Table S2.

The method limit of detection (LOD) was 5 ng L^{-1} in terms of ΣMC equivalents. A matrix-matched calibration curve (constructed in matrix-free lake water) was used for quantification, using spikes of a MC mixture before oxidation. We participated in a recreational water interlaboratory proficiency study with Abraxis/Eurofins. The determined ΣMC concentrations in the split samples analyzed by the in-house method was within ±30% of the consensus value.

[1] Wu, X., Xiao, B., Li, R., Wang, Z., Chen, X., & Chen, X. (2009). Rapid quantification of total microcystins in cyanobacterial samples by periodate-permanganate oxidation and reversed-phase liquid chromatography. Analytica Chimica Acta, 651(2), 241-247.

[2] Munoz, G., Duy, S. V., Roy-Lachapelle, A., Husk, B., & Sauvé, S. (2017). Analysis of individual and total microcystins in surface water by on-line preconcentration and desalting coupled to liquid chromatography tandem mass spectrometry. Journal of Chromatography A, 1516, 9-20.

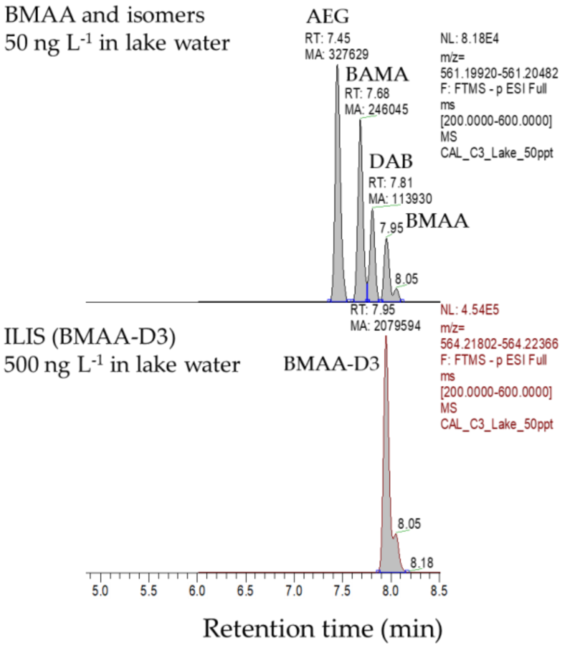

Figure S1. UHPLC-HRMS chromatograms of FMOC-derivatized BMAA isomers (AEG, BAMA, BMAA, and DAB) spiked at 50 ng L^{-1} in blank lake water. The lower pane shows the corresponding isotope-labeled internal standard (ILIS: BMAA-D3) spiked at 500 ng L^{-1}.

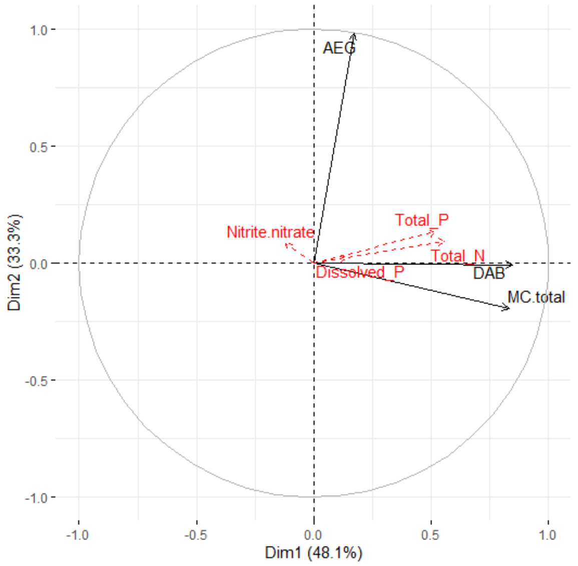

Figure S2. Principal component analysis (PCA) correlation circle of active variables (total MCs, AEG, DAB) and additional environmental variables superimposed on the plot (red font dotted arrows) for the PLSF site. The PCA is applied to a subset of n = 64 samples (two outliers removed). Chlorophyll-a data were not available for all time points and therefore could not be included in the statistical analysis.

Table S1. Details on the UHPLC-HRMS instrumental method for the analysis of FMOC-derivatized BMAA and its isomers.

<table>
<tr><th>Instrument</th><th colspan="3">Thermo Q-Exactive Orbitrap mass spectrometer
Dionex Ultimate 3400 UHPLC chain</th></tr>
<tr><td>Ionization</td><td colspan="3">Electrospray ionization source, negative ion mode</td></tr>
<tr><td>Acquisition mode</td><td colspan="3">Full Scan MS (m/z 200-600)</td></tr>
<tr><td>Analytical column</td><td colspan="3">Thermo Hypersil Gold C18 column (100 × 2.1 mm; 1.9 μm)</td></tr>
<tr><td>Column Temperature</td><td colspan="3">35°C</td></tr>
<tr><td>Analytical Mobile Phases</td><td colspan="3">A: 2.5 mM ammonium acetate in HPLC-water
B: acetonitrile</td></tr>
<tr><td rowspan="9">UHPLC gradient program</td><td>Time (min)</td><td>% B</td><td>Flow rate (μL/min)</td></tr>
<tr><td>0.0</td><td>25</td><td>450</td></tr>
<tr><td>2.0</td><td>25</td><td>450</td></tr>
<tr><td>4.0</td><td>40</td><td>450</td></tr>
<tr><td>8.0</td><td>50</td><td>450</td></tr>
<tr><td>8.4</td><td>95</td><td>450</td></tr>
<tr><td>9.4</td><td>95</td><td>450</td></tr>
<tr><td>9.5</td><td>25</td><td>450</td></tr>
<tr><td>10</td><td>25</td><td>450</td></tr>
<tr><td>Injection Volume</td><td colspan="3">1000 μL (on-line SPE)</td></tr>
<tr><td>On-line SPE column</td><td colspan="3">Thermo HyperSep Retain PEP column (20 mm × 2.1 mm, 40–60 μm)</td></tr>
<tr><td>On-line SPE Mobile Phases</td><td colspan="3">A: HPLC-water
B: acetonitrile</td></tr>
<tr><td rowspan="7">On-line SPE gradient program</td><td>Time (min)</td><td>% B</td><td>Flow rate (μL/min)</td></tr>
<tr><td>0.0</td><td>0</td><td>1500</td></tr>
<tr><td>2.0</td><td>0</td><td>1500</td></tr>
<tr><td>2.1</td><td>100</td><td>1500</td></tr>
<tr><td>6.9</td><td>100</td><td>1500</td></tr>
<tr><td>7.0</td><td>0</td><td>1500</td></tr>
<tr><td>10</td><td>0</td><td>1500</td></tr>
<tr><td>Source/gas parameters</td><td colspan="3">Sheath gas flow rate 55
Aux gas flow rate 10
Sweep gas flow rate 0
Spray voltage (kV) -3.5
Capillary temperature (°C) 350
Vaporizer temperature (°C) 400
S-lens RF level 60</td></tr>
<tr><td>Orbitrap parameters</td><td colspan="3">Resolution setting of 70,000 FWHM at m/z 200</td></tr>
</table>

		AGC target 3e6<br>Maximum Inject Time (ms) 100	
**Data processing**		*m/z*	Retention time (min)
	AEG (FMOC)	561.20201	7.45
	BAMA (FMOC)	561.20201	7.68
	BMAA (FMOC)	561.20201	7.95
	DAB (FMOC)	561.20201	7.81
	D3-BMAA (FMOC)	564.22084	7.95

Table S2. Details on the UHPLC-MS/MS instrumental method for the analysis of total microcystins via oxidative cleavage (MMPB method).

**Instrument**	**Thermo TSQ Quantiva triple quadrupole mass spectrometer**<br>**Accela UHPLC chain**		
**Ionization**	Electrospray ionization source, negative ion mode		
**Acquisition mode**	Selected reaction monitoring (SRM mode)		
**Analytical column**	Thermo Hypersil Gold C18 column (50 × 2.1 mm; 1.9 μm)		
**Column Temperature**	50°C		
**Analytical Mobile Phases**	A: HPLC-water<br>B: methanol		
**UHPLC gradient program**	Time (min)	% B	Flow rate (μL/min)
	0.0	60	500
	2.0	60	500
	4.2	100	500
	5.6	100	500
	5.7	60	500
	6.0	60	500
**Injection Volume**	1000 μL (on-line SPE)		
**On-line SPE column**	Thermo HyperSep Retain PEP column (20 mm × 2.1 mm, 40–60 μm)		
**On-line SPE Mobile Phases**	A: HPLC-water + 0.5% formic acid<br>B: methanol + 0.5%formic acid		
**On-line SPE gradient program**	Time (min)	% B	Flow rate (μL/min)
	0.0	0	1000
	2.0	0	1000
	2.1	100	1500
	4.1	100	1500
	4.2	0	1500
	6.0	0	1500
**Source/gas parameters**	Sheath gas pressure 35 a.u.<br>Aux gas pressure 15 a.u.<br>Sweep gas pressure 0 a.u.<br>Spray voltage (kV) -2.8<br>Ion transfer tube temperature (°C) 350<br>Vaporizer temperature (°C) 400		
**MS/MS acquisition parameters**	Dwell time 30 ms<br>Q1 and Q3 operated at a resolution of 0.7 Da FWHM<br>Q2 collision gas (CID) pressure (mTorr) 1.5<br>Tube lens (V) 43<br>Collision energy (V) 12		

	MS/MS transition	Retention time (min)
MMPB	207 → 131	2.90
D3-MMPB	210 → 131	2.90

Table S3. Measured concentrations (ng L^{-1}) of AEG, BAMA, BMAA, and DAB in freshwater lakes and reservoirs samples, with or without amendment of 0.1M TCA.

	**AEG (ng L^{-1})**		**BAMA (ng L^{-1})**		**BMAA (ng L^{-1})**		**DAB (ng L^{-1})**	
	**Without TCA**	**With TCA**	**Without TCA**	With TCA	Without TCA	With TCA	Without TCA	With TCA
TCA-test_SW-001	ND	ND	ND	ND	ND	ND	177	163
TCA-test_SW-002	31	92	ND	53	ND	ND	54	55
TCA-test_SW-003	42	52	ND	52	ND	ND	51	53
TCA-test_SW-004	ND	ND	ND	ND	ND	ND	ND	ND
TCA-test_SW-005	ND	12	ND	ND	ND	ND	ND	81
TCA-test_SW-006	ND	ND	ND	ND	ND	ND	ND	ND
TCA-test_SW-007	ND	6	ND	ND	ND	ND	ND	20
TCA-test_SW-008	ND	ND	ND	ND	ND	ND	ND	ND
TCA-test_SW-009	12	8	ND	ND	ND	ND	ND	21
TCA-test_SW-010	ND	15	ND	ND	ND	ND	ND	21
TCA-test_SW-011	ND	225	ND	ND	ND	ND	ND	ND
TCA-test_SW-012	ND	ND	ND	ND	ND	ND	ND	31
TCA-test_SW-013	ND	ND	ND	ND	ND	ND	ND	21
TCA-test_SW-014	ND	55	ND	ND	ND	ND	ND	18
TCA-test_SW-015	27	64	ND	ND	ND	ND	ND	28
TCA-test_SW-016	ND	7	ND	ND	ND	ND	ND	ND
TCA-test_SW-017	ND	ND	ND	ND	ND	ND	ND	24
TCA-test_SW-018	ND	15	ND	45	ND	ND	ND	19
TCA-test_SW-019	27	43	ND	ND	ND	ND	ND	20
TCA-test_SW-020	ND	ND	ND	ND	ND	ND	ND	20
TCA-test_SW-021	ND	21	ND	ND	ND	ND	ND	21
TCA-test_SW-022	ND	34	ND	ND	ND	ND	ND	ND
TCA-test_SW-023	ND	ND	ND	ND	ND	ND	ND	26
TCA-test_SW-024	ND	518	ND	ND	ND	ND	ND	ND
TCA-test_SW-025	ND	ND	ND	ND	ND	ND	ND	20
TCA-test_SW-026	ND	ND	ND	ND	ND	ND	ND	ND
TCA-test_SW-027	ND	ND	ND	ND	ND	ND	ND	ND
TCA-test_SW-028	ND	ND	ND	ND	ND	ND	43	70
TCA-test_SW-029	ND	ND	ND	ND	ND	ND	46	51
TCA-test_SW-030	ND	ND	ND	ND	ND	ND	51	66
TCA-test_SW-031	ND	34	ND	ND	ND	ND	ND	ND
TCA-test_SW-032	ND	72	ND	ND	ND	ND	ND	ND
TCA-test_SW-033	ND	ND	ND	ND	ND	ND	ND	ND
TCA-test_SW-034	ND	ND	ND	ND	ND	ND	47	52
TCA-test_SW-035	ND	ND	ND	ND	ND	ND	51	67
TCA-test_SW-036	ND	ND	ND	ND	ND	ND	ND	ND
TCA-test_SW-037	ND	ND	ND	29	ND	ND	ND	32
TCA-test_SW-038	ND	ND	ND	ND	ND	ND	85	64
TCA-test_SW-039	ND	ND	ND	ND	ND	ND	ND	53
TCA-test_SW-040	ND	35	ND	ND	ND	ND	ND	ND
TCA-test_SW-041	ND	ND	ND	ND	ND	ND	ND	ND
TCA-test_SW-042	ND	ND	ND	ND	ND	ND	ND	ND

TCA-test_SW-043	ND	ND	ND	ND	ND	ND	ND	ND
TCA-test_SW-044	ND	ND	ND	ND	ND	ND	ND	ND
TCA-test_SW-045	ND	ND	ND	ND	ND	ND	ND	ND
TCA-test_SW-046	ND	ND	ND	ND	ND	ND	ND	109
TCA-test_SW-047	ND	ND	ND	ND	ND	ND	ND	ND
TCA-test_SW-048	ND	ND	ND	ND	ND	ND	34	45
TCA-test_SW-049	83	74	ND	ND	ND	ND	ND	ND
TCA-test_SW-050	ND	ND	ND	ND	ND	ND	ND	ND
TCA-test_SW-051	ND	ND	ND	ND	ND	ND	ND	ND
TCA-test_SW-052	ND	ND	ND	ND	ND	ND	178	208
TCA-test_SW-053	ND	ND	ND	ND	ND	ND	ND	45
TCA-test_SW-054	ND	ND	ND	ND	ND	ND	ND	ND
TCA-test_SW-055	ND	10	ND	ND	ND	ND	ND	55
TCA-test_SW-056	ND	ND	ND	ND	ND	ND	32	45
TCA-test_SW-057	ND	ND	ND	ND	ND	ND	ND	ND
TCA-test_SW-058	ND	ND	ND	ND	ND	ND	19	27
TCA-test_SW-059	ND	ND	ND	ND	ND	ND	ND	ND
TCA-test_SW-060	ND	ND	ND	ND	ND	ND	ND	ND
TCA-test_SW-061	ND	ND	ND	ND	ND	ND	ND	ND
TCA-test_SW-062	51	78	ND	ND	ND	ND	35	50
TCA-test_SW-063	ND	33	ND	ND	ND	ND	ND	ND
TCA-test_SW-064	ND	37	ND	ND	ND	ND	ND	ND
TCA-test_SW-065	ND	ND	ND	ND	ND	ND	ND	ND
TCA-test_SW-066	ND	39	ND	ND	ND	ND	ND	15
TCA-test_SW-067	ND	ND	ND	ND	ND	ND	72	255
TCA-test_SW-068	ND	ND	ND	ND	ND	ND	ND	ND
TCA-test_SW-069	47	ND	ND	ND	ND	ND	75	52
TCA-test_SW-070	81	432	ND	ND	ND	ND	139	117
TCA-test_SW-071	22	99	ND	16	ND	ND	ND	37
TCA-test_SW-072	18	144	ND	50	ND	ND	ND	ND
TCA-test_SW-073	64	233	57	43	ND	ND	86	111
TCA-test_SW-074	ND	58	21	ND	ND	ND	49	61
TCA-test_SW-075	ND	38	ND	ND	ND	ND	32	66
TCA-test_SW-076	57	71	ND	33	ND	ND	56	59
TCA-test_SW-077	24	100	ND	53	ND	ND	62	85
TCA-test_SW-078	35	62	ND	53	ND	ND	34	95
TCA-test_SW-079	ND	49	ND	ND	ND	ND	ND	ND
TCA-test_SW-080	76	158	ND	38	ND	ND	112	81
TCA-test_SW-081	69	131	ND	33	ND	ND	79	83
TCA-test_SW-082	ND	75	ND	31	ND	ND	ND	66
TCA-test_SW-083	28	94	ND	56	ND	ND	ND	64
TCA-test_SW-084	22	71	ND	53	ND	ND	43	59
TCA-test_SW-085	45	192	57	53	ND	ND	73	119
TCA-test_SW-086	29	54	ND	24	ND	ND	49	55
TCA-test_SW-087	59	172	59	54	ND	ND	87	78
TCA-test_SW-088	ND	ND	ND	ND	ND	ND	11	27

TCA-test_SW-089	ND	ND	ND	ND	ND	ND	ND	ND
TCA-test_SW-090	ND	ND	ND	ND	ND	ND	10	16
TCA-test_SW-091	ND	ND	ND	ND	ND	ND	ND	12
TCA-test_SW-092	ND	ND	ND	ND	ND	ND	ND	ND
TCA-test_SW-093	3591	18814	ND	ND	ND	ND	ND	ND
TCA-test_SW-094	ND	37	ND	33	ND	ND	ND	57
TCA-test_SW-095	ND	ND	ND	ND	ND	ND	133	136
TCA-test_SW-096	ND	ND	ND	ND	ND	ND	ND	ND
TCA-test_SW-097	5	35	ND	52	ND	ND	ND	ND
TCA-test_SW-098	ND	35	ND	ND	ND	ND	ND	ND
TCA-test_SW-099	ND	ND	ND	ND	ND	ND	ND	27
TCA-test_SW-100	ND	58	ND	ND	ND	ND	ND	ND
TCA-test_SW-101	ND	ND	ND	ND	ND	ND	ND	ND
TCA-test_SW-102	ND	ND	ND	ND	ND	ND	ND	ND
TCA-test_SW-103	ND	11	ND	ND	ND	ND	ND	30
TCA-test_SW-104	ND	16	ND	ND	ND	ND	ND	33
TCA-test_SW-105	15	20	ND	ND	ND	ND	39	27
TCA-test_SW-106	ND	ND	ND	ND	ND	ND	43	49
TCA-test_SW-107	5	43	ND	ND	ND	ND	ND	52
TCA-test_SW-108	ND	ND	ND	ND	ND	ND	ND	ND
TCA-test_SW-109	ND	34	ND	ND	ND	ND	ND	53
TCA-test_SW-110	ND	ND	ND	ND	ND	ND	ND	ND
TCA-test_SW-111	ND	ND	ND	ND	ND	ND	ND	ND
TCA-test_SW-112	ND	ND	ND	ND	ND	ND	ND	ND
TCA-test_SW-113	ND	33	ND	ND	ND	ND	ND	ND
TCA-test_SW-114	ND	ND	ND	ND	ND	ND	ND	18
TCA-test_SW-115	ND	ND	ND	ND	ND	ND	25	32
TCA-test_SW-116	ND	ND	ND	ND	ND	ND	113	237
TCA-test_SW-117	ND	ND	ND	ND	ND	ND	10	58
TCA-test_SW-118	ND	ND	ND	ND	ND	ND	ND	29
TCA-test_SW-119	ND	34	ND	ND	ND	ND	330	359
TCA-test_SW-120	28	65	ND	ND	ND	ND	ND	ND
TCA-test_SW-121	ND	ND	ND	ND	ND	ND	12	22
TCA-test_SW-122	ND	ND	ND	ND	ND	ND	14	30
TCA-test_SW-123	ND	ND	ND	45	ND	ND	42	39
TCA-test_SW-124	ND	ND	ND	ND	ND	ND	41	35
TCA-test_SW-125	139	133	ND	53	ND	ND	78	105
TCA-test_SW-126	ND	ND	ND	ND	ND	ND	16	32
TCA-test_SW-127	ND	ND	ND	ND	ND	ND	79	181
TCA-test_SW-128	ND	ND	ND	ND	ND	ND	107	171
TCA-test_SW-129	ND	35	ND	ND	ND	ND	ND	ND
TCA-test_SW-130	ND	ND	ND	ND	ND	ND	ND	ND
TCA-test_SW-131	ND	ND	ND	ND	ND	ND	46	45
TCA-test_SW-132	ND	ND	ND	ND	ND	ND	20	32
TCA-test_SW-133	ND	51	ND	ND	ND	ND	13	201
TCA-test_SW-134	ND	49	ND	55	ND	ND	89	87

TCA-test_SW-135	ND	ND	ND	ND	ND	ND	ND	ND
TCA-test_SW-136	ND	ND	ND	ND	ND	ND	ND	53
TCA-test_SW-137	ND	34	ND	ND	ND	ND	ND	ND
TCA-test_SW-138	ND	ND	ND	ND	ND	ND	ND	ND
TCA-test_SW-139	ND	ND	ND	ND	ND	ND	ND	ND
TCA-test_SW-140	ND	ND	ND	ND	ND	ND	ND	56
TCA-test_SW-141	ND	ND	ND	ND	ND	ND	ND	ND
TCA-test_SW-142	ND	ND	ND	ND	ND	ND	24	88
TCA-test_SW-143	ND	178	ND	ND	ND	ND	81	230
TCA-test_SW-144	ND	ND	ND	ND	ND	ND	ND	ND
TCA-test_SW-145	ND	7	ND	ND	ND	ND	ND	ND
TCA-test_SW-146	ND	ND	ND	ND	ND	ND	ND	ND
TCA-test_SW-147	ND	ND	ND	ND	ND	ND	ND	ND
TCA-test_SW-148	ND	ND	ND	ND	ND	ND	ND	ND
TCA-test_SW-149	ND	ND	ND	ND	ND	ND	ND	ND
TCA-test_SW-150	ND	ND	ND	ND	ND	ND	610	1261
TCA-test_SW-151	ND	ND	ND	ND	ND	ND	45	46
TCA-test_SW-152	ND	7	ND	ND	ND	ND	ND	19
TCA-test_SW-153	ND	ND	ND	ND	ND	ND	ND	35
TCA-test_SW-154	ND	ND	ND	ND	ND	ND	ND	28
TCA-test_SW-155	5	ND	ND	ND	ND	ND	ND	23
TCA-test_SW-156	28	20	ND	ND	ND	ND	ND	36
TCA-test_SW-157	ND	6	ND	ND	ND	ND	ND	23
TCA-test_SW-158	ND	ND	ND	ND	ND	ND	32	39

ND: Non-detect data. Limits of detection were 5 ng L^{-1} for AEG and 10 ng L^{-1} for BAMA, BMAA, and DAB.
